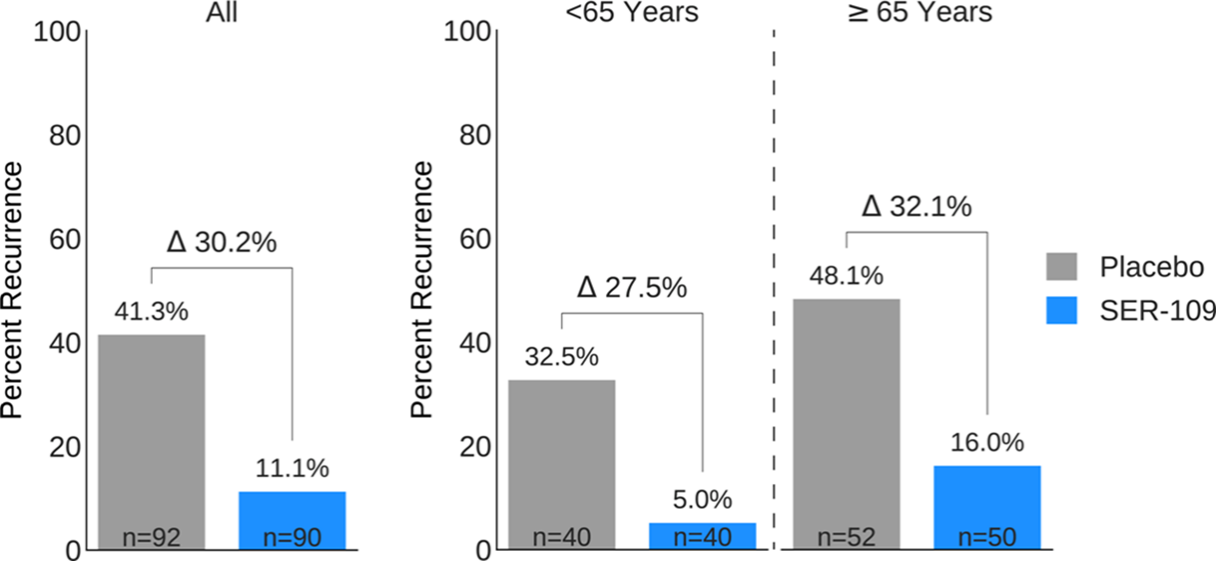# Efficacy and Safety of Investigational Microbiome Drug SER-109 for Treatment of Recurrent *Clostridioides difficile* Infection

**DOI:** 10.1017/ash.2021.10

**Published:** 2021-07-29

**Authors:** Barbara McGovern, Mathew Sims, Colleen Kraft, Elaine Wang, Kelly Brady, Christopher Ford, O’Brien Edward, Mary-Jane Lombardo, Jennifer Wortman, Kevin Litcofsky, Jennifer Mahoney, Christopher McChalicher, Jonathan Winkler, Sarah Garant, John Aunins, Matthew Henn, Lisa von Moltke

## Abstract

**Background:** Antibiotics targeted against *Clostridioides difficile* bacteria are necessary, but insufficient, to achieve a durable clinical response because they have no effect on *C. difficile* spores that germinate within a disrupted microbiome. ECOSPOR-III evaluated SER-109, an investigational, biologically derived microbiome therapeutic of purified Firmicute spores for treatment of rCDI. Herein, we present the interim analysis in the ITT population at 8 and 12 weeks. **Methods:** Adults ≥18 years with rCDI (≥3 episodes in 12 months) were screened at 75 US and CAN sites. CDI was defined as ≥3 unformed stools per day for <48 hours with a positive C. difficile assay. After completion of 10–21 days of vancomycin or fidaxomicin, adults with symptom resolution were randomized 1:1 to SER-109 (4 capsules × 3 days) or matching placebo and stratified by age (≥ or <65 years) and antibiotic received. Primary objectives were safety and efficacy at 8 weeks. Primary efficacy endpoint was rCDI (recurrent toxin+ diarrhea requiring treatment); secondary endpoints included efficacy at 12 weeks after dosing. **Results:** Overall, 287 participants were screened and 182 were randomized (59.9% female; mean age, 65.5 years). The most common reason for screen failure was a negative *C. difficile* toxin assay. A significantly lower proportion of SER-109 participants had rCDI after dosing compared to placebo at week 8 (11.1% vs 41.3%, respectively; relative risk [RR], 0.27; 95% confidence interval [CI], 0.15–0.51; p-value <0.001). Efficacy rates were significantly higher with SER-109 vs placebo in both stratified age groups (Figure 1). SER-109 was well-tolerated with a safety profile similar to placebo. The most common treatment-emergent adverse events (TEAEs) were gastrointestinal and were mainly mild to moderate. No serious TEAEs, infections, deaths, or drug discontinuations were deemed related to study drug. **Conclusions:** SER-109, an oral live microbiome therapeutic, achieved high rates of sustained clinical response with a favorable safety profile. By enriching for Firmicute spores, SER-109 achieves high efficacy while mitigating risk of transmitting infectious agents, beyond donor screening alone. SER-109 represents a major paradigm shift in the clinical management of patients with recurrent CDI. Clinicaltrials.gov Identifier NCT03183128. These data were previously presented as a late breaker at American College of Gastroenterology 2020.

**Funding:** Seres Therapeutics

**Disclosures:** None

**Figure 1.**